# Unmet Needs in the Care of Patients with Duchenne Muscular Dystrophy in Brazil

**DOI:** 10.1055/s-0046-1817019

**Published:** 2026-03-23

**Authors:** Alexandra Prufer de Queiroz Campos Araujo, Andre Vinícius Soares Barbosa, Michele Michelin Becker, Andressa Araujo Braga, Marcela Câmara Machado Costa, Alulin Tácio Quadros Santos Monteiro Fonseca, Marcondes Cavalcante França Junior, Juliana Gurgel Giannetti, Vanessa Van Der Linden, Carlos Alberto da Silva Magliano, André Luiz Santos Pessoa, Edmar Zanoteli

**Affiliations:** 1 Universidade Federal do Rio de Janeiro, Faculdade de Medicina, Departamento de Pediatria, Rio de Janeiro RJ, Brazil.; 2 Hospital Infantil João Paulo II, Belo Horizonte MG, Brazil.; 3 Santa Casa de Belo Horizonte, Belo Horizonte MG, Brazil.; 4 Hospital de Clínicas de Porto Alegre, Departamento de Pediatria, Porto Alegre RS, Brazil.; 5 Instituto Nacional de Cardiologia, Núcleo de Avaliação de Tecnologias em Saúde, Rio de Janeiro RJ, Brazil.; 6 Escola Bahiana de Medicina e Saúde Pública, Salvador BA, Brazil.; 7 Universidade Federal de São Paulo, Escola Paulista de Medicina, Departamento de Neurologia e Neurocirurgia, São Paulo SP, Brazil.; 8 Universidade Estadual de Campinas, Faculdade de Ciências Médicas, Departamento de Neurologia, Campinas SP, Brazil.; 9 Universidade Federal de Minas Gerais, Faculdade de Medicina, Departamento de Pediatria, Belo Horizonte MG, Brazil.; 10 Hospital Maria Lucinda, Recife PE, Brazil.; 11 ASAS Valor em Saúde, Rio de Janeiro RJ, Brazil.; 12 Hospital Infantil Albert Sabin, Fortaleza CE, Brazil.; 13 Universidade Federal do Ceará, Fortaleza CE, Brazil.; 14 Universidade de São Paulo, Faculdade de Medicina, Departamento de Neurologia, São Paulo SP, Brazil.

**Keywords:** Muscular Dystrophy, Duchenne, Standard of Care, Health Inequities, Rare Diseases, Healthcare Disparities, Health Care Quality, Access, and Evaluation

## Abstract

**Background:**

Duchenne muscular dystrophy is a rare, progressive neuromuscular disorder primarily affecting boys, and it follows a predictable course. Early intervention is essential for effective management, but disparities in the care of patients with rare diseases hinder access to optimal treatment.

**Objective:**

To identify unmet needs and challenges in the care of patients with Duchenne muscular dystrophy within the Brazilian public health system compared with the private system.

**Methods:**

A cross-sectional observational study using the Delphi method was conducted with ten neurologists specialized in Duchenne muscular dystrophy. The specialists participated in rounds of surveys to reach consensus on key issues, including diagnosis, treatment, and care. Data was analyzed using descriptive statistics.

**Results:**

According to the Delphi panel, the public health system had an average diagnostic delay of 25 months compared with 10 months in the private sector. Although genetic testing is critical, it is not funded by the public health system. Other barriers included delayed corticosteroid treatment, limited access to multidisciplinary care, and insufficient medical devices. Patients in the public health system lost ambulation earlier (11–12 years of age) than those in the private sector (13–14 years of age). Life expectancy was significantly shorter in the public system, averaging 19 to 20 years compared with 26 to 27 years of age in the private sector.

**Conclusion:**

There are significant disparities in the care of patients with Duchenne muscular dystrophy within Brazil's public health system, resulting in worse outcomes. Enhancing access to genetic testing and early multidisciplinary care is crucial to improve the quality of life and survival of these patients.

## INTRODUCTION

**Figure 1:**
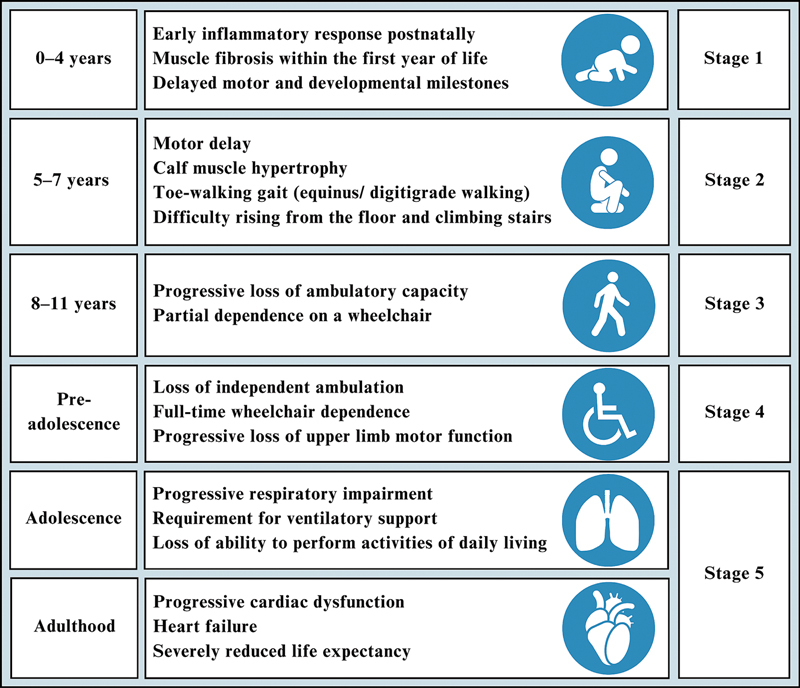
Notes: Stage 1: asymptomatic phase; stage 2: early ambulatory phase; stage 3: late ambulatory (transitional) phase; stage 4: early non-ambulatory phase; stage 5: late non-ambulatory phase. Age ranges are approximated and may vary by individual progression. Stages of Duchenne muscular dystrophy (DMD). ^4–10^

Duchenne muscular dystrophy (DMD) is a rare X-linked recessive neuromuscular disease that primarily affects boys and initially presents with developmental delays and elevated serum creatine kinase levels. 1 The disease follows a relative predictable trajectory ( Figure 1 ), progressing from loss of ambulation to upper-limb limitations, respiratory dysfunction, and cardiomyopathy, reducing life expectancy. 2

Effective management of DMD requires early intervention, guided by established international 3 4 5 and Brazilian 6 7 8 protocols. Healthcare disparities, including variations in expertise and multidisciplinary requirements, pose barriers to optimal DMD care, 9 often exacerbated by differences in healthcare settings and funding mechanisms. 9 10

The current study aimed to elucidate the specific challenges encountered in diagnosing and treating DMD patients within the Brazilian Unified Health System (Sistema Único de Saúde, SUS, in Portuguese) compared with the private healthcare sector. By identifying critical unmet needs, the research intends to contribute to improve the care provided to DMD patients.

## METHODS

The present cross-sectional study used the Delphi method with a convenience sample of 10 neurologists selected according to predefined criteria: active practice in SUS outpatient clinics (with the option of also working in the private sector), management of at least 6 DMD patients per year, and a minimum of 1 year of specialization. All specialists invited agreed to participate, met the predefined inclusion criteria, and are coauthors of this research.

Two Delphi rounds were conducted during June and July 2024, with consensus *a priori* defined as ≥ 70% of agreement or disagreement. The first round consisted of a semi-structured online questionnaire (Supplementary Material) distributed via Survey Monkey (SurveyMonkey Inc.). At the beginning of the second round, an in-person session with half the panel was conducted to validate and refine the statements derived from the first-round results. The second round then proceeded with a questionnaire (Supplementary Material) using a five-point Likert scale. Both rounds were completed anonymously to minimize peer influence. Aggregated results were shared with the panel after each round, and topics without consensus were qualitatively analyzed to capture divergent expert perspectives.

**Figure 2:**
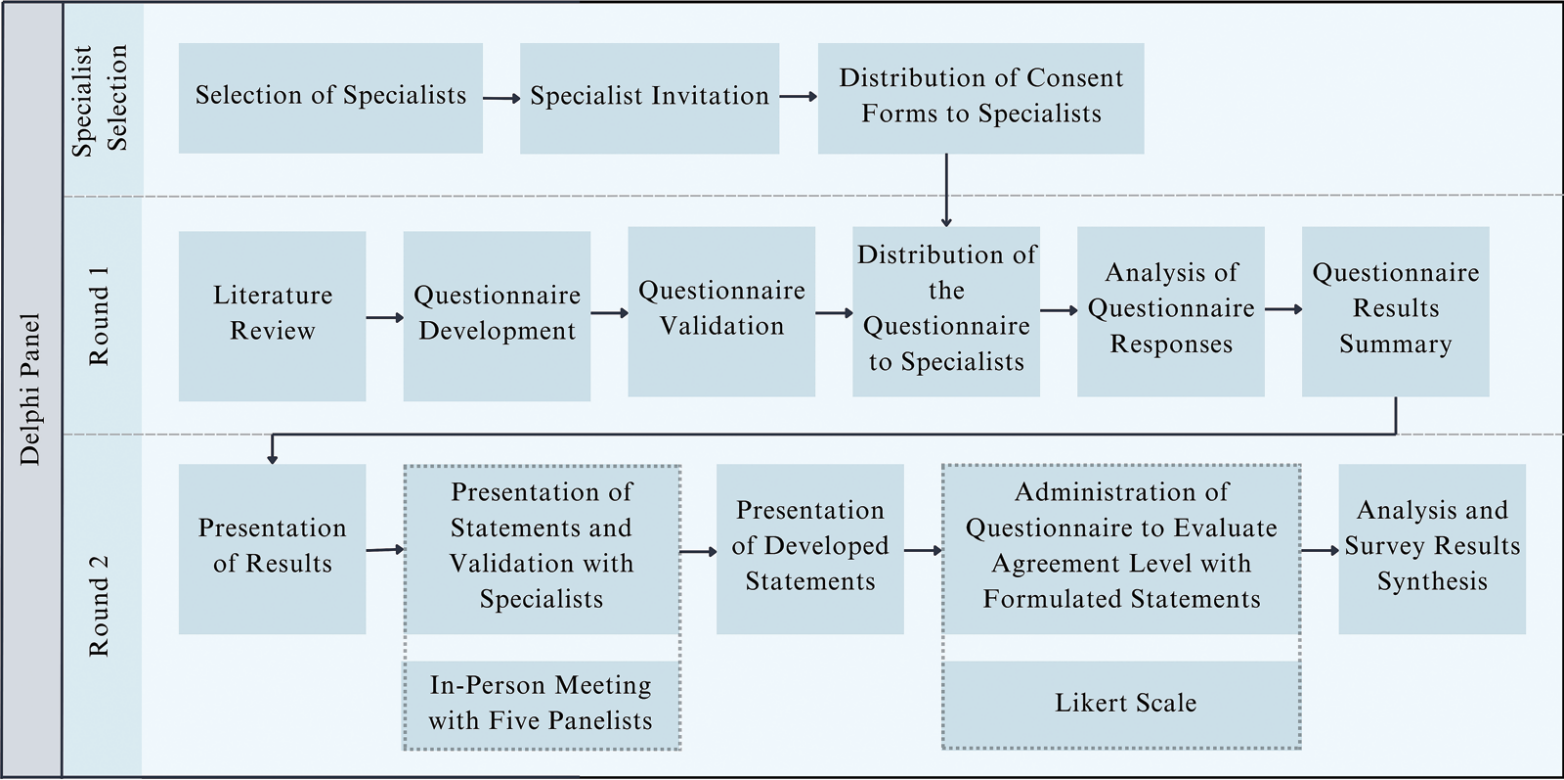
Delphi panel steps.

The questionnaires were developed following a rapid literature review and validated by independent physicians to ensure clarity and completeness. The overall process ( Figure 2 ), including the invitation, informed consent, questionnaire development, and data collection and analysis, was independently managed by the principal researchers without interference from external entities to minimize bias. A detailed description of the methodological process—including search strategies, questionnaire development, bias mitigation, and the consensus framework—is provided in the Supplementary Material (available at https://www.arquivosdeneuropsiquiatria.org/wp-content/uploads/2025/11/ANP-2025.0171-Supplementary-Material.docx ).

Quantitative data were analyzed using descriptive statistics (mean, median and frequencies). First-round data were presented in non-overlapping numerical intervals. Regarding the Likert scale, responses of “strongly agree” and “agree” were counted as agreement, while “disagree” and “strongly disagree” were counted as disagreement, enabling the calculation of agreement or disagreement percentages. Data analysis was performed using the Excel (Microsoft Corp.) software.

### Ethical considerations

The proposed research followed the guidelines of Resolutions n°. 510/2016 and n°. 466/12 of the Brazilian National Health Council. 11 12

Notably, registration and evaluation of data collection by the CEP/CONEP system were not required, as the study was considered a theoretical analysis of spontaneously-occurring situations in professional practice, without disclosure of identifiable data. 12

## RESULTS

### Specialist profile

**Figure 3:**
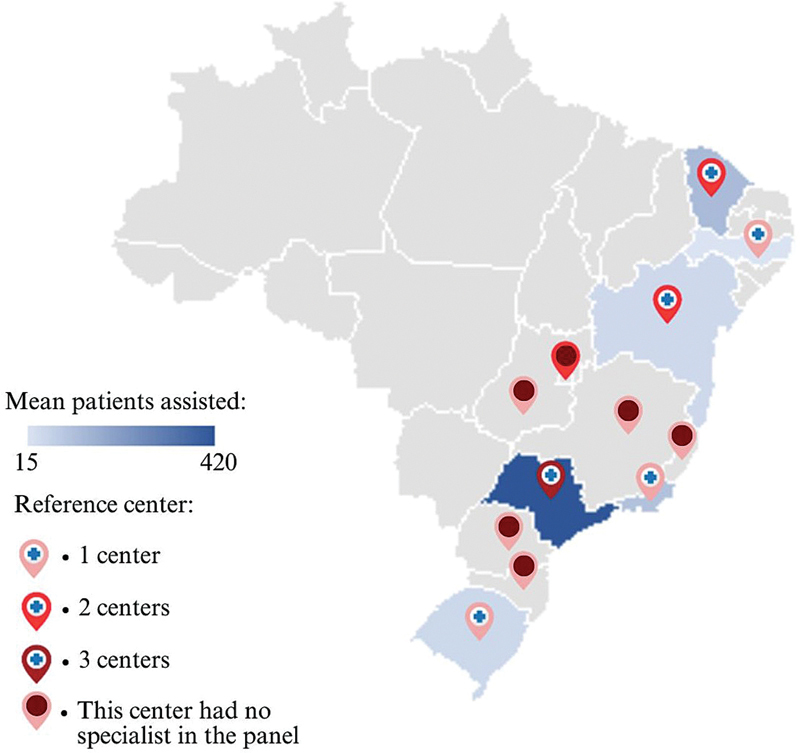
Geographic regions of Brazil and average number of patients treated by the Delphi panel specialists. **Notes:** Mean number of Duchenne muscular dystrophy (DMD) patients assisted by each specialist in the Delphi panel; reference center: Reference Center for Rare Diseases in Brazil. 22

Ten specialists participated in both rounds of the Delphi panel ( Table 1 ), representing different regions of practice ( Figure 3 ).

**Table 1:** Delphi specialist profile

Description	Results
**Specialty: n (%)**	
Pediatric neurologist	60% (6)
Neurologist	40% (4)
**Mean years of medical practice**	26.4 ± 7.9
**Mean number of Duchenne muscular dystrophy patients treated by the specialist**	110 ± 75.7
**Region of practice: n (%)**	
South	10% (1)
Southeast	60% (6)
Northeast	30% (3)
**Specialist's outpatient care type: n (%)**	
Outpatient care in the public system	100% (10)
Outpatient care in the private system	70% (7)

Survey results are summarized in Table 2 , covering diagnosis, treatment, follow-up, medical devices, complications, and caregiver support.

**Table 2:** Delphi panel consensus summary

**Delphi panel consensus Summary**	Diagnosis is significantly delayed in the SUS, leading to later treatment initiation compared with the private sector.
	Genetic testing is necessary to confirm the diagnosis of the disease; however, it remains non-reimbursable by the SUS.
	Treatment with glucocorticoids starts later for SUS patients, potentially contributing to an earlier loss of ambulation and exacerbation of kyphoscoliosis.
	Multidisciplinary follow-up is less frequent in the SUS, leading to increased complications and faster disease progression.
	Medical technologies are insufficiently available in the SUS, leading to earlier complications and reduced quality of life.
	Surgery for kyphoscoliosis correction is recommended in the public and private systems, but longer waiting times in the SUS often result in contraindications as the patients' conditions worsen.
	Full-time caregiving becomes essential from stage 4, placing a significant burden on families.
	Complications, including loss of ambulation and vertebral or long-bone fractures manifest earlier in SUS patients, potentially attributable to delays in diagnosis and suboptimal care.
	Transitioning care for DMD patients from pediatric to adult services poses significant challenges, impacting long-term disease management.

Abbreviations: DMD, Duchenne Muscular Dystrophy; SUS, Sistema Único de Saúde (Brazilian Unified Health System).

Note: Consensus defined as agreement ≥ 70%.

### Diagnosis

The mean age at diagnosis for DMD patients in the SUS was higher than in the private sector. The diagnosis process in the SUS was protracted (100% of agreement), with patients waiting more than 25 months from the first symptom to diagnosis, compared with just 10 months in the private sector. All specialists agreed that molecular testing (next-generation sequencing [NGS] or multiplex ligation-dependent probe amplification [MLPA]) is necessary to confirm the diagnosis of the disease. Although genetic testing is not reimbursed by the SUS, it was performed in more than 80% of the patients treated by the specialists in public health involved in the Delphi panel.

### Treatment

Regarding treatment, most specialists (90%) consider deflazacort the ideal glucocorticoid due to its favorable safety profile. However, it is not reimbursed by the SUS, which, instead, uses prednisone or prednisolone ( Table 3 ).

**Table 3:** Delphi panel statements

Statement	Agreement rate*
The waiting time from the observation of the first signs and symptoms to the definitive diagnosis is longer in public health system patients than in private health system patients.	100%
Deflazacort, prednisone, or prednisolone may be prescribed for DMD treatment, but deflazacort presents fewer adverse effects, particularly weight gain.	100%
The most common glucocorticoid treatment in the public health system is prednisone or prednisolone, as deflazacort is not available.	100%
Reference center professionals conduct regular evaluations and generally have expertise in neuromuscular disease, unlike local care providers.	100%
Patients in advanced stages (4 and 5) show lower adherence to follow-up and treatment.	90%
The main reasons for low adherence are mobility challenges and costs, including transportation and caregiver absenteeism.	100%
Invasive or non-invasive ventilation for more than 15 hours may be indicated for stage-5 patients, but these devices are not available for home use in the public health system.	78%
The time required for orthosis creation and the materials used in the public health system are not always ideal.	100%
Wheelchairs should be individually adapted to DMD patients, but these adaptations are rarely available in the public health system.	100%
Heart failure tends to occur earlier in the public health system patients compared with private health system patients.	33%**
Death occurs at a younger age in public health system patients compared with private health system patients.	86%

Abbreviation: DMD, Duchenne muscular dystrophy.

Notes: *Agreement rate: percentage of specialists who agreed or strongly agreed with the statement. *No consensus; consensus was reached if 70% of the specialists either agreed or strongly agreed, or conversely, disagreed or strongly disagreed.

Glucocorticoid therapy is recommended for all patients starting from stage 2 (100% of agreement), ideally beginning at the mean age of 2 to 4 years (90% of agreement). This early start is observed in 71% of the patients treated by the Delphi panel specialists in the private system ( Table 4 ), while treatment in the SUS is usually delayed (80% of agreement). The specialists emphasized that delays in treatment initiation can result in an earlier loss of ambulation, occurring before the growth spurt phase, thereby exacerbating kyphoscoliosis.

**Table 4:** Average age at diagnosis and at the start of the treatment of the patients cared for by the specialists

Age of the patients (years)		Private health system specialists (N = 7): n (%) (n/7)	Public health system specialists (N = 10): n (%)
**Age at diagnosis**	≤ 2	1 (14)	0
	3–4	2 (29)	0
	5–6	3 (43)	3 (30)
	7–8	1 (14)	7 (70)*
**Age at the start of the treatment**	2–4	5 (71)*	1 (10)
	5	1 (14)	1 (10)
	6–8	1 (14)	8 (80)*

Notes: Ten specialists work in the public health system, and seven specialists also work in the private health system; *consensus.

### Multidisciplinary follow-Up

Follow-up care by neurologists should occur every 4 to 6 months (90–100% of agreement). However, SUS patients usually receive only 1 to 2 consultations per year (100% of agreement). The profile of patients followed by the Delphi specialists shows that 60 to 69% of those treated in the SUS reside in different municipalities.

Other specialists, such as pediatricians, cardiologists, pulmonologists, ophthalmologists, orthopedists, gastroenterologists, nephrologists, and endocrinologists, are available in the SUS, but less frequently than needed (88–100% of agreement; Supplementary Material).

Multidisciplinary follow-up, including motor and respiratory physiotherapy, speech therapy, psychotherapy, occupational therapy, and nutrition, should ideally increase in frequency as the disease progresses (Supplementary Material). These services, however, are insufficiently available or accessible to many patients, particularly within the SUS, resulting in suboptimal follow-up frequency and significantly impacting disease progression (100% of agreement). The reduced frequency of multidisciplinary care is attributed to a scarcity of specialists and socioeconomic challenges, including mobility issues, transportation costs, and caregiver absenteeism, especially in stages 4 and 5 (90–100% of agreement).

Occupational therapy was identified as the least-available specialty (100% of agreement), with weekly or biweekly sessions provided to less than 30% of patients treated by Delphi panel specialists.

The availability of qualified professionals in the SUS and in the private sector is limited, contributing to increased osteoarticular and cardiorespiratory complications and accelerating disease progression, as observed by the specialists. Professionals in reference centers typically provide periodic evaluations and possess expertise in neuromuscular diseases. In contrast, those who routinely manage patients closer to their residences, whether in the SUS or private healthcare systems, often lack this specialized expertise (100% of agreement).

### Complementary tests

Complementary tests, such as blood tests, X-rays, echocardiograms, electrocardiograms, polysomnography, and pulmonary function tests, are more frequently requested in the later stages (4 and 5) of the disease (Supplementary Material). Although these tests are available in the SUS, access varies significantly by location, and waiting times can be longer, as observed by the Delphi panel specialists.

### Medical devices and other technologies

According to the specialists, DMD patients require various technologies throughout each stage of the disease (Supplementary Material). Orthoses are recommended from stage 2 (90% of agreement), and manual wheelchairs, from stage 3 (90% of agreement). Although electric wheelchairs are advised at stage 5, they are rarely provided by the SUS (80% of agreement). While orthoses and wheelchairs are available, they are often inadequate in quality and customization (100% of agreement). According to the Delphi panel specialists, this inadequacy negatively affects posture and exacerbates kyphoscoliosis.

The manual resuscitator bag is used to support the inspiratory phase of manually-assisted coughing, whereas mechanical cough assistance requires a specific device. Manually-assisted coughing and mechanical cough assistance devices are recommended starting at stage 4, but they are not provided by the SUS (100% of agreement). Bilevel positive airway pressure (BiPAP) devices are recommended from stage 4 and are occasionally offered to patients (89% of agreement). In contrast, invasive ventilation for more than 15 hours is frequently recommended from stage 5 (70% of agreement) but is not available (78% of agreement). Home care becomes necessary at stages 4 or 5 (90% of agreement); yet, it is not available in the SUS (100% of agreement). Specialists note that the unavailability of these devices can lead to earlier respiratory and osteoarticular complications, worsening quality of life, and increasing caregiver burden.

### Caregiver

Patients require full-time caregiver support beginning at stage 4 (90% of agreement).

### Education and employment

Patients with DMD usually attend school up to stage 3 (80% of agreement). School attendance declines primarily due to inadequate infrastructure, lack of transportation, and insufficient trained professionals (100% of agreement). According to the Delphi panel specialists, only 19 to 26% of the patients are estimated to potentially enter the job market. Cognitive issues and workplace accessibility present significant barriers to employment (100% of agreement).

### Neuropsychiatric comorbidities

According to the panel specialists, DMD patients present many neuropsychiatric comorbidities, such as intellectual disability (26–35%), anxiety (28–36%), depression (26–35%), global developmental disorder (20–29%), attention deficit hyperactivity disorder (23–32%), dyslexia (10–16%), oppositional defiant disorder (6–11%), and obsessive-compulsive disorder (5–10%).

### Complications of Duchenne muscular dystrophy

According to the Delphi panel, the loss of ambulation is the most frequent complication, occurring earlier in SUS patients (100% of agreement), at a mean age of 11 to 12 years, compared with 13 to 14 years in the private sector. Vertebral or long-bone fractures also occur earlier in SUS patients (71% of agreement). However, there was no consensus regarding the timing of heart failure: 67% of the specialists disagreed that it occurs earlier in SUS patients compared with those in the private sector.

While surgery for kyphoscoliosis correction is recommended for patients in the public and private systems, it is more commonly performed in the private system (30–39% of the patients) compared with the SUS (12–17% of the patients). The waiting times for surgery in the SUS are significantly longer (100% of agreement), ranging from 22 to 27 months, compared with 4 to 8 months in the private system, often leading to contraindications for SUS patients by the time the surgery is authorized (100% of agreement).

Annual hospitalizations occur in ∼20% of the patients in stage 4 and in nearly 50% of those in stage 5, as estimated by Delphi panel specialists, similarly in both systems (90% of agreement).

### Transition of care

Transitioning from pediatric to adult care is a significant challenge for DMD patients, affecting continuity of care as they reach adulthood (90% of agreement).

### Mortality

Patients with DMD often die from heart failure (100% of agreement). According to the panel specialists, heart failure accounts for 80% of deaths in the private system and 50% in the SUS, while pneumonia is responsible for 20% in the private system and 30% in the SUS. The mean age at death is estimated to be 26 to 27 years in the private system and 19 to 20 years in the SUS, indicating that deaths occur earlier in SUS patients (86% of agreement).

## DISCUSSION

There are significant unmet needs in the journey of Brazilian DMD patients in the SUS. Disparities between public and private healthcare begin early, at the stages of suspicion and diagnosis. In the SUS, patients face delays due to differences in system organization and limited availability of specialist and genetic testing. 13 Unlike the private practice, in which families can directly access specialists, SUS patients must go through a referral network to reach specialized centers, often located in larger cities. 14 Referral pathways and geographic barriers hinder timely diagnosis and care. 15 16 Access disparities are evident nationwide, with some regions entirely lacking reference centers 17 ( Figure 2 ).

While diagnostic delays are common in rare diseases, 18 early detection and simple screening tools remain crucial to improve patient outcomes. 19 20 Ideally, diagnosis should occur in early childhood, to enable timely genetic counseling and access to multidisciplinary care. 3 4 5 6 7 8 However, after an accurate diagnosis, patients often encounter limited access to resources and specialized treatment. 14

Unfortunately, missed opportunities for early intervention negatively affect the course of DMD. Restricted access to medical devices in the SUS may contribute to earlier loss of ambulation, as well as orthopedic, respiratory, and cardiac complications at younger ages. This may result in life expectancy for DMD patients in the SUS being nearly a decade shorter than in countries with better access to ventilatory support (median: 29.9 years) and comparable to pre-1990 levels without ventilatory support (median: 19 years). 21

While advancements in DMD care have been made globally, with earlier intervention delaying disease progression and improving life expectancy, 3 4 5 6 7 8 21 22 23 the Brazilian healthcare system has also made progress but still faces significant challenges. Although individual disease trajectories vary due to genetic heterogeneity, factors like early rehabilitation, timely corticosteroid use, and regular ventilatory and cardiac assessments remain essential for better outcomes. 24

Although 76% of Brazilians rely exclusively on the SUS, 25 more than half of national health expenditure comes from private sources, reinforcing a 2-tiered structure that undermines the system's universality. 26 27 Over the past decades, the SUS has expanded coverage and improved health outcomes; however, access to specialized care remains limited, with long waiting times and marked regional disparities, including unequal distribution of equipment, infrastructure, and human resources. 27 28 29 30 Despite universal coverage, 3.8% of Brazilians reported unmet needs for health services, and 7.5%, for medications in 2019, with inequalities concentrated among poorer populations and the Northern region. 31 Financial barriers and persistent racial, spatial, and income inequalities further restrict access to high-complexity care, which remains concentrated in wealthier regions. 30 31 These structural inequities directly affect the management of complex health conditions, 27 28 29 32 such as DMD. This disparity in rare-disease care is not unique to Brazil and remains a significant challenge globally. 13 33 34 In Brazil, national policies such as the 2014 National Policy for Comprehensive Care for People with Rare Diseases 35 have advanced equitable access within the SUS; however, significant barriers persist. Addressing these issues requires expanding public resources, prioritizing key patient needs, improving access to diagnosis and treatment, enhancing healthcare-worker training, strengthening system infrastructure, standardizing clinical guidelines, and fostering patient involvement in care decisions. 14 16 33 36

The current study provides valuable insights into the challenges faced by DMD patients within the SUS, but some limitations should be noted. The sample of 10 specialists covered 3 regions of the country, representing 83% of the population, 37 but the findings may not be fully generalizable to underrepresented areas. Notably, the specialists represent all regions with available DMD reference centers, except the Midwest. 17 Additionally, focusing solely on specialist perspectives may overlook the experiences of patients and caregivers, which are essential to understand the unmet needs.

Future research on DMD care in Brazil should incorporate perspectives from patients, caregivers, and allied health professionals to provide a more comprehensive understanding of the challenges and unmet needs in DMD care.

The present study highlights the significant disparities in the care of DMD patients between Brazil's public and private health systems. The findings reveal delays in diagnosis, limited access to appropriate treatments, and the unavailability of essential medical devices in the SUS, all of which contribute to poorer patient outcomes, including earlier loss of ambulation and reduced life expectancy. Addressing these inequities requires improving access to genetic testing, ensuring timely interventions, and providing comprehensive multidisciplinary care to enhance patient outcomes and quality of life.
